# Mechanisms of T cell-mediated antitumor immunity within tertiary lymphoid structures

**DOI:** 10.3389/fimmu.2026.1883563

**Published:** 2026-07-20

**Authors:** Yuqin Wang, Wenlong Cao, Min Wu, Hui Wang

**Affiliations:** 1 Department of Gynecologic Oncology, Women’s Hospital, Zhejiang University School of Medicine, Hangzhou, China; 2 Zhejiang Key Laboratory of Precision Diagnosis and Therapy for Major Gynecological Diseases, Women’s Hospital, Zhejiang University School of Medicine, Hangzhou, China; 3 Department of Nephrology, Union Hospital, Tongji Medical College, Huazhong University of Science and Technology, Wuhan, China

**Keywords:** antitumor immunity, immunotherapy, T cell subsets, tertiary lymphoid structures, tumor microenvironment

## Abstract

Tertiary lymphoid structures (TLS) are ectopic lymphoid-like organs formed under chronic inflammatory stimulation, which have been increasingly recognized as indicators of favorable clinical prognosis and enhanced immunotherapy response in multiple solid tumors. T cells are essential constituents of TLS, involved in their formation, maintenance, and immune function. They exhibit substantial heterogeneity in quantity, phenotype, and spatial localization across different tumor types. Follicular helper T (Tfh) cells act as a central subset in TLS by promoting B cell activation, germinal center (GC) development, and antibody production. They have been widely regarded as key predictors of favorable therapeutic response and prolonged survival. In addition, peripheral helper T (Tph) cells, regulatory T (Treg) cells, and follicular regulatory T (Tfr) cells also perform immunomodulatory functions within TLS. Their effects can either enhance or suppress antitumor responses, depending on the tumor type, TLS spatial distribution, and maturation status. This review examines the phenotypic heterogeneity, functional fate, and intercellular interactions of T cells within TLS. It also explores the immunoregulatory role of TLS and their potential in novel immunotherapy strategies. Further research should aim to the evolution process of immune populations within TLS and standardized model establishment, in order to accelerate their clinical translation in precision immunotherapy.

## 1 Introduction

TLS have recently become a frontier hotspot in tumor microenvironment (TME) research, offering novel biological perspective and investigative strategies for cancer immunotherapy. TLS are node-like structures that form in non-lymphoid organs under conditions of chronic inflammation or antigen stimulation, resembling secondary lymphoid organs (SLOs) in morphology and function. TLS are not preset tissues like SLOs development, but are inducible immune aggregates formed under pathological conditions.

The study of TLS can be traced back to the 1960s. In 1964, Ziff was the first to identify lymphoid-like aggregates in the synovium of rheumatoid arthritis patients, suggesting a potential role in localized immune responses (1). In 1992, Picker and Butcher introduced the terms “tertiary lymphoid organs” (TLOs) or “tertiary lymphoid tissues” (TLTs) to characterize non-encapsulated lymphocyte aggregates that arise locally during chronic inflammatory processes (2). In 2001, the term TLS was officially established to describe lymphoid clusters with GC–like features found in rheumatoid synovitis (3). In recent years, with the in-depth study of cancer immunity, Giraldo et al. firstly linked the presence of TLS to the efficacy of immune checkpoint inhibitors (ICIs) in 2015 (4). This association subsequently confirmed in clear cell renal cell carcinoma (ccRCC) (5), melanoma (6), and head and neck squamous cell carcinoma(HNSCC) (7), highlighting clinical relevance of TLS in cancer immunity.

Structurally, TLS are composed of T cells, B cells, dendritic cells (DCs), high endothelial venules (HEVs), and fibroblast-like cells, forming spatially organized T and B cell zones. B cells are the core components of TLS, especially in mature TLS, they can undergo somatic hypermutation, affinity maturation, immunoglobulin class switching, and plasma cell differentiation (5, 8, 9), thereby contributing to antibody-mediated immune responses. Studies have shown that patients with tumor tissues rich in B cells, plasma cells and mature TLS tend to have better survival prognosis and immunotherapy response rate (10–12), making TLS an important biomarker for predicting immune therapy. Compared with the immunogenic function of B cells, T cells in TLS exhibit more complex heterogeneity and functions. On the one hand, helper T cells (such as Tfh cells and Tph cells) can enhance humoral immunity and antitumor activity by promoting B cell response and germinal center formation. On the other hand, Treg and Tfr cells may restrain immune activation by secreting immunosuppressive cytokines. Therefore, the composition and activation state of T cells within TLS are critical determinants of their immune functionality and therapeutic outcomes.

Current studies suggest that the abundance, maturation status, spatial localization, and immune cell composition of TLS collectively determine their immunological role and prognostic value across cancer types. Based on their cellular composition and degree of organization, different TLS maturation stages have been reported in human tumors. Immature TLS (primary lymphoid follicles) are largely composed of a T cell zone and B cell follicle with DC but no germinal center (GC). Mature TLS (secondary lymphoid follicles) incorporate lymphatic vessels, a segregated T cell zone including T follicular helper (Tfh) cells, mature DC, and a B cell follicle that includes mantle and GC B cells (active GC contains proliferating B cells), follicular DC and macrophages (9, 13–15). Although prior studies have outlined the functional framework of TLS, the precise lineage, spatial organization, and intercellular interactions between T cells and other cells remain incompletely understood.

## 2 The fate and function of T cells in TA-TLS

**Figure 1:**
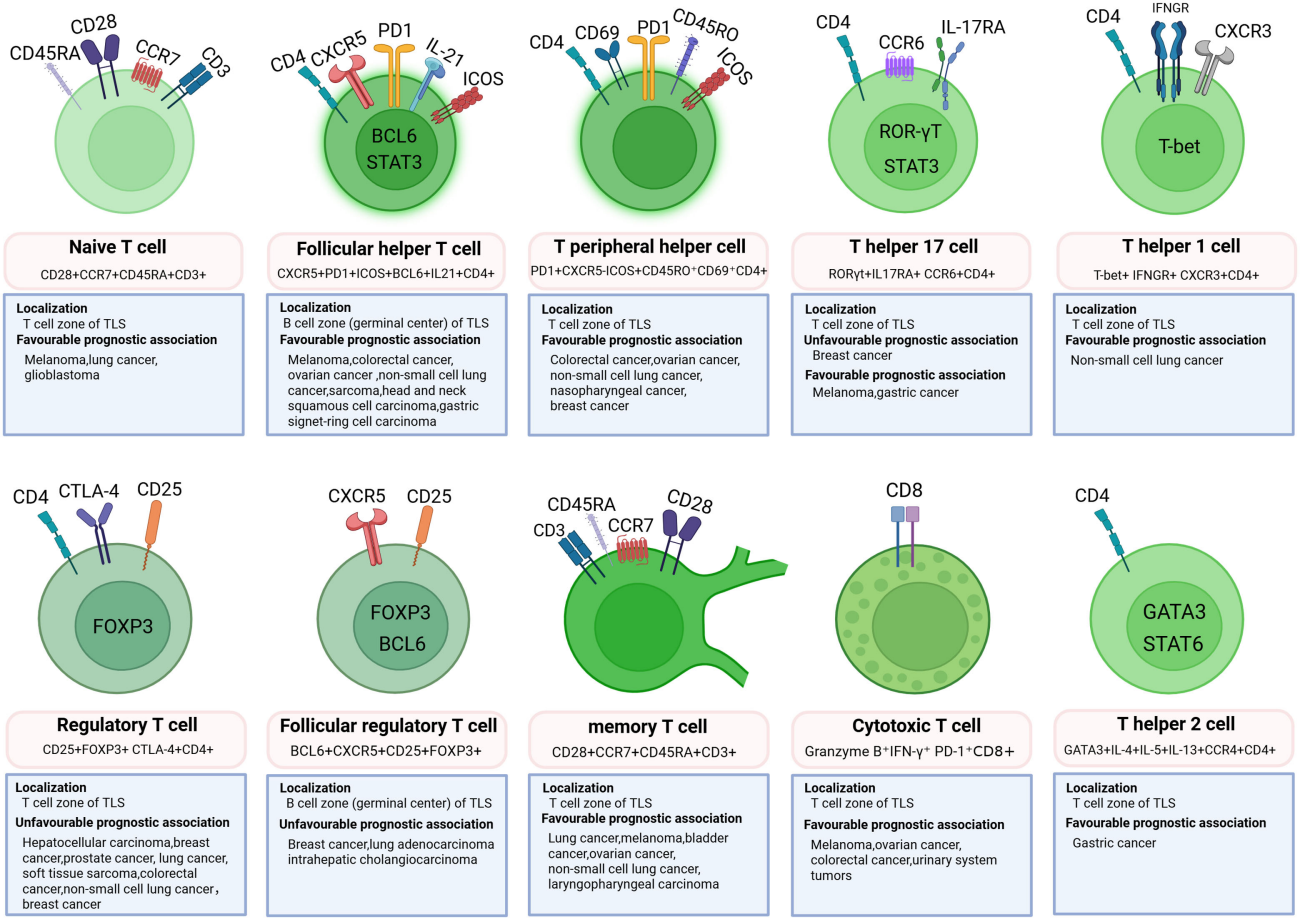
Heterogeneity of T cell subsets in tumor-associated tertiary lymphoid structures (TA-TLS). This schematic illustrates the differentiation trajectories, phenotypic markers, spatial localization, prognostic associations, and representative tumor types of major T cell subsets identified within TA-TLS. Naive CD4^+^ T cells differentiate into multiple specialized subsets, including Tfh, Tph, Th1, Th2, Th17, Treg and Tfr cells, each with distinct transcriptional regulators (e.g., BCL6, T-bet, RORγt, FOXP3, GATA3). CD8^+^ T cells include cytotoxic T lymphocytes (CTLs) and memory T cells, with Trm cells also indicated. Prognostic impacts (favorable or unfavorable) vary by subset and cancer type, as summarized from recent literature. This figure highlights the functional and phenotypic diversity of T cells within TLS and their context-dependent roles in antitumor immunity. Detailed descriptions of each subset and associated references are provided in Section 2. Tfh, follicular helper T; Tph, peripheral helper T; Th1, T helper 1; Th2, T helper 2; Th17, T helper 17; Treg, regulatory T; Tfr, follicular regulatory T; Trm, tissue-resident memory T.

In recent years, developments in multiplex immunohistochemistry, single-cell RNA sequencing (scRNA-seq), and spatial transcriptomics have allowed researchers to map different T cell subsets in tumor-associated TLS (TA-TLS). These T cell populations have been widely reported in tumors and other conditions such as autoimmune diseases and organ transplantation. It is noteworthy that the presence and infiltration levels of T cells display marked heterogeneity. In most tumor types, such as melanoma, lung cancer, and glioblastoma, the frequency of naive CD4^+^ or CD8^+^ T cells is very low (16–18) (**Figure 1**). This observation aligns with the characteristic of TLS as an antigen-experienced T cell enrichment region. However, in solid tumors such as colorectal cancer (CRC), ovarian cancer (OC), and non-small cell lung cancer (NSCLC), enrichment of functional helper T cells including Tfh and Tph cells reflects the heterogeneity in TLS maturation and functional states (19–21) (**Figure 1**). In addition, Treg and Tfr cells are subsets of immunosuppressive T cells, commonly observed in TLS. These cells can produce cytokines including IL-10, IL-35, and TGF-β, attenuating antitumor immune responses and correlating with poor prognosis (22–25). Therefore, a systematic understanding of the heterogeneity and function of T cells in TLS is of great significance for elucidating their immunoregulatory mechanisms and developing precise immunotherapeutic strategies.

### 2.1 Follicular helper T cells

**Figure 2:**
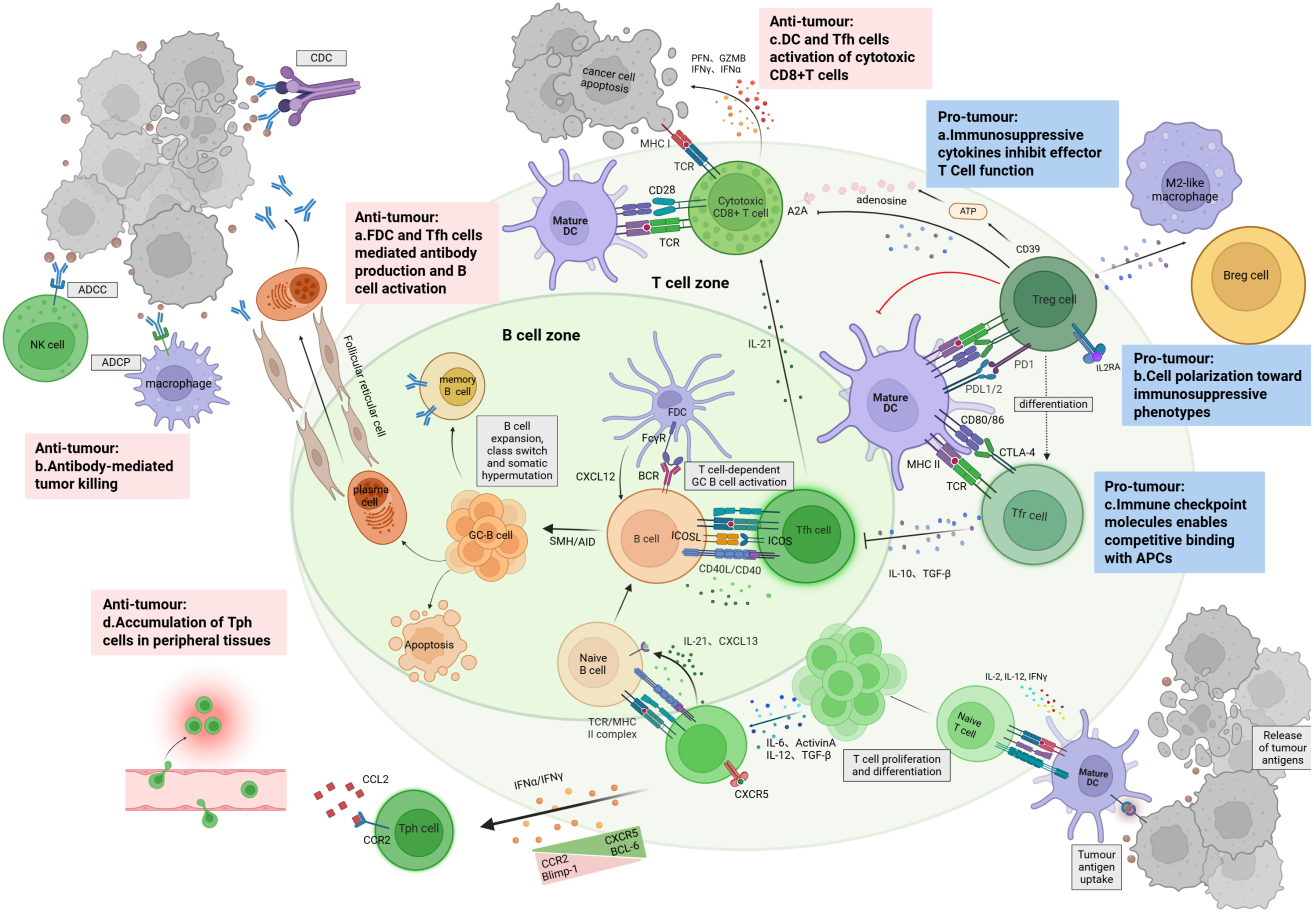
Immunological functions of T cell subsets within TLS architecture. This diagram depicts the spatial organization and functional interactions of T cells within the T cell zone and B cell zone of a mature TLS. Key anti-tumor mechanisms include: **(a)** antibody production and B cell activation mediated by FDCs and Tfh cells; **(b)** ADCC and ADCP involving NK cells and macrophages; **(c)** activation of cytotoxic CD8^+^ T cells by cDCs and Tfh cells; and **(d)** accumulation of Tph cells in peripheral tissues contributing to TLS formation and B cell recruitment. Pro-tumor mechanisms include the secretion of immunosuppressive cytokines (e.g., IL-10, TGF-β) by Tregs that inhibit effector T cell function. Key molecular interactions (e.g., CD40L/CD40, TCR/MHC-II, IL-21, CXCL13, CXCR5) and signaling pathways are indicated. This figure integrates structural and functional features of TLS discussed in Section 2 and Section 3.FDCs, follicular dendritic cells; ADCC, antibody-dependent cellular cytotoxicity; ADCP, antibody-dependent cellular phagocytosis; cDCs, conventional dendritic cells; Tph,T peripheral helper; Tregs, regulatory T cells.

With a typical phenotype of CD4^+^CXCR5^+^PD-1^+^ICOS^+^Bcl6^+^IL-21^+^, Tfh cells are essential to the T cell zone in TLS (**Figure 1**). Of note, the presence and abundance of Tfh cells are closely linked to TLS maturation status. They are located at the periphery of the follicular region and are one of the important markers of mature TLS (26–32). In contrast, immature TLS are characterized by a paucity of Tfh cells and a predominance of stromal cells and lymphocytes. These cells secrete chemokines including CXCL13 and IL-7, thereby recruiting lymphoid tissue-inducing (LTi) cells (B cells, Th17 cells, and M1 macrophages) to the tumor site, which initiates the early stages of TLS development (33–35). The transition from immature to mature TLS is accompanied by a progressive accumulation of Tfh cells. Their differentiation is driven by naïve CD4^+^ T cells in response to antigen stimulation and co-stimulatory signals such as ICOS/ICOSL, IL-12, IL-23, and TGF-β (36, 37). Through IL-4 and IL-21 secretion, Tfh cells assist B cell in affinity maturation, GC formation, and isotype switching (**Figure 2**). Bcl6 governs Tfh cells fate by downregulating PSGL-1 and CCR7 while upregulating CXCR5 and PD-1, promoting Tfh cells migration to the follicular region and guiding B cells recruitment through CXCL13 (38–50). The spatial co-localization of Tfh cells and B cells in TLS reflects active antitumor immunity and favorable therapeutic responses in cancer (51–54).

The assistance of Tfh cells to B cells depends on a variety of co-stimulatory pathways, including ICOS-ICOSL, CD40-CD40L, and CD30-CD30L (55). IL-21 produced by Tfh cells is critical for B cell maturation and antibody affinity maturation, and also enhances CD8^+^ T cell antitumor function, as demonstrated in chronic viral infection models and human NSCLC tissues (32, 56–61) (**Figure 2**). This mechanism has been demonstrated in chronic viral infections. By activating STAT3 and BATF pathways and coordinating IRF4, IL-21 boosts CD8^+^ T cell function and prevents exhaustion (62–64). Furthermore, Tfh cells can assist in the initial activation of CD8^+^ T cells under the condition of spatial proximity of DC, CD4^+^ and CD8^+^T cells through the “DC bridge” mechanism (65, 66). The fate and function of Tfh cells are regulated by PD-1 signaling. Persistent PD-1 signaling impairs Tfh cells maintenance and IL-21 secretion, while immune checkpoint inhibitors (ICIs) may enhance Tfh-mediated B cells activation and antibody production by blocking the PD-1 pathway (67–69).

The crosstalk between Tfh cells and exhausted CD8^+^ T (Tex) cells has also attracted attention. Multiplex immunohistochemistry indicates co-localization of Tfh and Tex cells within TLS (70). Tex cells-derived CXCL13 recruits Tfh and CXCR5^+^ B cells into TLS, promoting their structural maturation (71). Meanwhile, IL-21 secreted by Tfh cells inhibits the terminal exhaustion of Tex cells, sustaining their effect (60, 72–74). Notably, CXCL13^+^CD8^+^ Tex cells can predict immunotherapy response (75), and also drive early TLS formation via the CXCL13 axis, forming a positive feedback (70).

Nonetheless, Tfh cells may exert immunosuppressive effects by inducing Tregs recruitment or differentiating into Tfr cells in autoimmune and chronic inflammatory conditions (76), highlighting the need for analyzing of Tfh function.

### 2.2 T peripheral helper cells

Tph cells, originally identified as PD-1^high^CXCR5**^—^**CD4^+^T cells in rheumatoid arthritis. They can recruit B cells by secreting CXCL13 and support humoral immunity, despite lacking the canonical Tfh marker CXCR5 (77–80). In rheumatic diseases, the number of Tph cells is positively correlated with disease activity, suggesting a pathogenic role in chronic inflammation (78, 81, 82). Tph cells typically exhibit a terminally differentiated effector or memory phenotype characterized by CD3^+^CD45RO^+^CD69^+^CD4^+^CD27^−^CCR7^−^, indicating tissue residency and continuously stimulating B cells in inflammatory environment (77) (**Figure 1**).

Functionally, Tph cells support B cells responses by producing IL-21, ICOS, Maf, and SLAM family proteins like SLAMF1 and SLAMF5 (79). Compared to Tfh cells, Tph cells have significantly lower expression levels of Bcl-6 and higher expression levels of Blimp-1, likely limiting their role in GC (83) (**Figure 2**). Regarding the origin of Tph cells, using lineage-tracing approaches in human lymph nodes, Alcazar et al. demonstrated that Tph cells share substantial TCR clonal overlap with Tfh cells, indicating a common clonal origin (84). Tph cells may originate from the phenotypic remodeling of Tfh cells in a specific inflammatory environment. The Bcl-6/Blimp-1 antagonistic axis plays a central role in this conversion process. Bcl-6 governs Tfh cell fate by promoting CXCR5 expression and GC homing, while Blimp-1 antagonizes Bcl-6 and restricts Tfh differentiation, thereby promoting non-Tfh effector fates (27, 85).Under the continuous stimulation by inflammatory cytokines, some Tfh cells may downregulate CXCR5 and acquire the ability to migrate to non-lymphoid tissues, transforming into Tph cells (84) (**Figure 2**). This process may allow Tph cells to seed inflamed tumor sites before TLS formation, initiating B cells activation and humoral responses.

Tph cells have recently been observed in ovarian, nasopharyngeal, and breast cancers (14, 36, 86) (**Figure 1**). In these solid tumors, Tph cells are typically clustered in the tumor stroma or TLS regions and expressed CXCL13, recruiting CXCR5^+^ B and Tfh cells to promote the formation and maturation of TLS. Importantly, higher Tph infiltration aligns with greater TLS formation, better immunotherapy responses and survival outcome. However, *in vitro* co-culture experiments of Tph cells and B cells from rheumatoid arthritis patients showed that Tph cells could promote memory B cell proliferation and activation, but could not induce class switching or high-affinity antibody production, which are notably exhibited in Tfh cells (87).Thus, Tph cells are considered to be key CD4^+^ T cells with follicular-like function in peripheral tissues, capable of promoting B cell responses and TLS neogenesis in the absence of lymphoid microenvironments. Tph cells act as potential initiators of humoral immunity in the tumor microenvironment. Enhancing function or stabilizing phenotype of Tph cells may offer a novel strategy to modulate TLS formation and improve antitumor immune responses in future therapies.

### 2.3 T helper 1/T helper 2/T helper 17 cells

T helper (Th) cells are the key effector T cells in TLS, which play a central role in maintaining local immune homeostasis and driving the initiation and maintenance of antitumor immunity. Th cells in TLS mainly include Th1, Th2, and Th17 subsets, which play different functions in different immune response pathways. Th cell s are activated and lineage polarized through interactions with DCs and B cells, which rely on co-stimulatory signals like CD40–CD40L, thereby enhancing B cell expansion, plasma cell differentiation and antibody isotype switching (88–90). The differentiation of Th cells is finely regulated by a variety of cytokines and chemokines in the TME. Specifically, IL-12, IL-18, and interferon-γ (IFNγ) induce Th1 cells generation; IL-4 and IL-13 promote Th2 differentiation; and IL-6, TGF-β, and IL-23 are critical for the generation of Th17 cells (91–93). These signals may originate from local antigen-presenting cells (APCs) or be secreted by tumor cells or tumor-associated stromal cells, reflecting the profound influence of the TME.

Within TLS, various Th cell subsets cooperate to regulate multi-level immune responses. Th1 cells activate cellular immune responses by secreting IFN-γ and enhancing the antigen presentation and cell-killing activity of macrophages, DCS and cytotoxic T cells (94, 95). Th1 cells often correlate with better immune infiltration and prognosis in tumors, suggesting that they may participate in antitumor immunity by promoting inflammatory response and tumor antigen recognition. In contrast, Th2 cells drive humoral immunity by releasing IL-4, IL-5, and IL-13, which enhance B cell proliferation and antibody secretion (96, 97). Th2 activity is often associated with antiparasitic, allergic, and antibody-mediated immunity, but its role may be immunosuppressive in some tumor types. Th17 cells, through IL-17A, IL-17F and IL-22 secretion, participate in neutrophil recruitment, tissue repair, and mucosal barrier maintenance, and exhibit complex and bidirectional roles in tumor immunity (97, 98). IL-17 promotes fibroblastic reticular cells (FRCs) proliferation and network stability, while triggering the secretion of CXCL13 or CCL19 and promoting the homing of T and B cells to TLS (99). In immature TLS, Th17 cells act as lymphoid tissue-inducing (LTi) cells and contribute to the initiation of TLS (100). However, in esophageal squamous cell carcinoma (ESCC), patients with a promising prognosis had mature TLSs, which are characterized by a high ratio of proliferative B cells and CD4 T cells, including memory B cells and Th17 cells (101). In the experimental autoimmune encephalomyelitis (EAE) mouse model, activated Th17 cells can convert into Tfh cells in TLS regions (102). The phenotypic plasticity of Th17 cells allows them to play different biological roles in different environments. For example, in murine breast cancer models, IL-17 has been reported to promote tumor growth and metastasis, indicating that Th17 cells may exert pro-tumor effects (103) (**Figure 1**). Therefore, the role of Th17 cells is highly bidirectional, requiring detailed analysis based on tumor type, developmental stage, and TLS evolution.

Moreover, there is a dynamic balance maintained between the functional state of Th and Treg cells. Treg cells secrete IL-10 and TGF-β to limit excessive Th cell activation, maintain immune tolerance, and prevent tissue damage (22, 104). The lineage fates of Th17 and Treg cells are mutually regulated by the transcription factors RORγt and FOXP3. RORγt drives Th17 differentiation, while FOXP3 inhibits it to induce the generation of Treg (105–107) (**Figure 1**). This transcriptional regulatory axis also plays an important role in tumor immunity, and the tilt of its balance may determine whether TLS are dominated by inflammatory or immunosuppressive responses. An in-depth understanding of Th1, Th2, Th17, and their interaction mechanisms with Treg cells may provide a theoretical foundation and practical strategies for targeting TLS function and enhancing cancer immunotherapy responses.

### 2.4 Regulatory T cells

Regulatory T cells (Tregs) are key immunosuppressive CD4^+^ T cell subsets in TLS. defined by high expression of CD25 and FOXP3 (**Figure 1**). Treg cells play a core role in maintaining immune tolerance, preventing autoimmunity and controlling inflammatory responses. However, they are frequently linked to immune evasion, antitumor immunosuppression, and poor treatment response in cancer (108–110). FOXP3-driven Tregs differentiation is regulated by cytokines like IL-2, TGF-β, and IL-10, which can originate from tumor cells, tumor-associated immune cells (108–110).

Within TLS, Tregs are mainly located in the T cell zone and form a local immune regulatory hotspot with APCs. One major mechanism involves Treg-expressed CD39 converting extracellular ATP into 5’-AMP, which is subsequently metabolized by CD73 into immunosuppressive adenosine. Adenosine binds to A2A receptors on DCs and T cells, inhibiting T cell proliferation and antigen-presenting functions, thereby establishing an immunosuppressive barrier within TLS (22, 23). Besides, Tregs employ several mechanisms to directly suppress effector T (Teff) and B cells (23, 110–114). First, Treg broadly inhibits T cell activation, B cell antibody production and APCs maturation by secreting inhibitory cytokines such as IL-10, TGF-β and IL-35. Second, by expressing immune checkpoint molecules, such as CTLA-4, PD-1 and LAG-3, which compete with APCs for binding and further limit co-stimulatory signaling. Third, Tregs downregulate the metabolic and functional status of peripheral immune cells depending on cell-contact (**Figure 2**).

The accumulation of Tregs in TA-TLS is strongly linked to local immunosuppression and unfavorable prognosis. Immature TLS typically mediate immunosuppressive effects accompanied by Treg infiltration, whereas in mature TLS, Tregs can exert more potent immunosuppressive effects through direct contact with Tfh and B cells (115). For example, in early-stage hepatocellular carcinoma (HCC), intratumoral TLS often have an immunosuppressive environment riched in IL-10 and TGF-β, suggesting that Tregs may shape a “cold” immune state unresponsive to immunotherapy (116). In breast, prostate, and lung cancers, enrichment of Treg cells is repeatedly confirmed to be related with anti-tumor therapy resistance and immune escape (23, 108, 117, 118). Recent studies have shown that Tregs may also contribute to tumor progression through the CXCL13–CXCR5 signaling. CXCL13 mediates the recruitment of suppressive cells like Tregs and myeloid-derived suppressor cells (MDSCs), which induce IL-10 production and form an immunosuppressive feedback that weakens antitumor response (119–122). More importantly, the immunosuppressive effect of Treg often counteracts the beneficial anti-tumor effect of CD8^+^ T cells in NSCLC (23), CRC (123), and soft tissue sarcoma (STS) (124) (**Figure 1**). Treg also regulates the formation of PNAd^+^ HEVs and indirectly affects the homing and recruitment of lymphocytes, especially naive T cells and memory T cells, further limiting immune mobilization (125, 126).

Notably, Tregs not only exert suppressive effects alone, but also often cooperate with regulatory B cells (Bregs) to sustain an immunosuppressive state within TLS. In breast cancer, the co-occurrence of Tregs and Bregs within TLS is associated with poor metastasis-free survival, marking them as indicators of immunosuppressive TLS (111). Animal experiments have confirmed Tregs as key modulators of TLS immune activity. For example, in KP genotype mouse models of lung cancer, Treg depletion alleviated local immunosuppression and enhanced the activity of CD8^+^ T cells and Th1 cells in TA- TLS, boosting antitumor immune response (22). This finding provides a strong rationale for targeting Tregs to activate TLS function and also offers a new direction for synergistic immunotherapy. In summary, Treg cells suppress antitumor immune responses and regulate T cell or B cell functions through multi-level mechanisms such as metabolic inhibition, cytokine modulation, checkpoint molecule expression, and helper cell migration. In the future, how to specifically target Tregs in TLS to relieve their suppressive function and avoid the loss of systemic immune tolerance will be an important research direction.

### 2.5 Follicular regulatory T cells

Tfr cells, a specialized subset of regulatory T cells, play a key role in modulating GC activity and shaping the intensity and specificity of humoral immunity. Tfr cells are uniquely marked by expression of FOXP3, BCL6, CXCR5, CD25, and GARP (**Figure 1**), enabling their migration to the follicular region in response to CXCL13 (127, 128). Tfr cells regulate the interaction between Tfh cells and B cells. Tfr cells compete with Tfh cells for cytokine IL-21 within the GC, thereby limiting the survival of Tfh cells and their ability to assist B cells (24, 129). TCR sequencing analysis showed that Tfr cells share a developmental lineage with classical Tregs, suggesting that Tfr cells may derive from thymic or peripherally induced Tregs rather than Tfh cells (117). Functionally, Tfr cells retain some traits of Tfh cells, such as GC tropism and limited expression of IL-21, while strongly overlapping with Tregs in expression of immunosuppressive molecules like CTLA-4 and GARP.

Tfr cells are largely restricted to mature TLS with well-developed GCs, as their recruitment depends on CXCR5 and CXCL13. In immature TLS, where GCs are absent, Tfr cells are rarely detected, underscoring their specialized role as GC regulators rather than general immunosuppressive cells. Tfr cells fine-regulate humoral immunity through several mechanisms, including: 1) limiting Tfh activation and clonal expansion in the GC; 2) restraining B cell somatic hypermutation and antibody class switching; 3) preventing the generation and amplification of autoreactive B cell clones; 4) modulating the timing and scope of GC responses to prevent excessive immunopathology (119, 122, 130). The differentiation mechanism of Tfr cells is closely related to that of Tfh cells. Both of them rely on co-stimulatory signals such as CD28 and ICOS to activate the expression of BCL6, enabling follicular localization and functional maturation (120, 130–132). However, Tfr cells must maintain FOXP3 expression and moderate expression of BCL6, which is a balance considered essential for Treg and Tfh cells. Studies have shown that under certain conditions, memory Tfh cells can convert into functional Tfr cells upon co-activation of IL-2, STAT3, and STAT5 signaling pathways (133). This process is IL-2 dose-dependent: low-doses of IL-2 promote the differentiation of Tfh into Tfr cells, whereas high doses strongly inhibit their generation (134, 135). This mechanism has been experimentally validated in SLOs.

Tfr cells highly express the immune checkpoint molecule CTLA-4, and their immune regulatory function may have an important impact on immunotherapy. On the one hand, Tfr cells block the costimulatory signal of CD80/CD86 on DCs surface through CTLA-4, limiting T cell activation and weakening the therapeutic effects of ICIs (136). On the other hand, Tfr cells show limited responsiveness to PD-1 blockade alone, but combining or sequencing anti-CTLA-4 treatment before PD-1 inhibition has been shown to enhance immune activity. It is suggested that Tfr may limit the therapeutic window of anti-PD-1 therapy through CTLA-4-dominated negative regulatory pathway (137) (**Figure 2**). Tfr cells may also be involved in the early immune organization and homeostatic regulation of TLS. For example, in primary Sjögren’s syndrome (pSS) patients, an increased Tfr/Tfh ratio is closely associated with TLS formation in salivary glands, suggesting a regulatory role for Tfr cells in fine-tuning TLS development (138). In IgG4-related sialadenitis, excessive Tfr cells accumulation in the GC is linked to abnormal GC morphology and increased IgG4^+^ plasma cells, suggesting a central role in abnormal follicular structure and humoral immunity (139). Although this phenomenon has not yet been widely confirmed in TA-TLS, it has attracted attention in related fields.

In breast cancer, studies have shown that Tfr cells suppress IL-21 and IFN-γ production through GARP-dependent TGF-β signaling mechanism, thereby inhibiting B cells activation and antibody production. The Tfh/Tfr ratio plays a key role in shaping TLS function. Tfh-dominant TLS show pro-inflammatory features with enhanced IgG secretion, B cell proliferation and CD8^+^ T cell responses, while Tfr-dominant TLS display an immunosuppressive phenotype (24, 140). Similar mechanisms have also been observed in other tumors. In microinvasive lung adenocarcinoma (LUAD), follicle-localized Tfr cells enrichment is correlated with low CD8^+^ T cell infiltration, suggesting that they may impair T cell-mediated antitumor immunity (141). In intrahepatic cholangiocarcinoma, Tfr cells accumulation in peritumoral TLS is strongly associated with worse prognosis, suggesting that it may inhibit anti-tumor immunity by regulating humoral immune balance (142) (**Figure 1**).

In summary, Tfr cells regulate Tfh function and B cell responses within TLS through their unique follicular localization and multi-mechanism immunosuppressive mechanisms. Their roles in the regulation of antibody production, follicular structural stability, and modulation of antitumor immunity are being gradually revealed. Future research are needed to further clarify the functional heterogeneity of Tfr cells across different tumor types and its intervention potential in immunotherapy, particularly for personalized strategies to its intervention potential in immunotherapy.

### 2.6 Memory T cells

In the TME, memory T cells, particularly the CD8^+^ subsets, are central effectors in sustaining antitumor immune responses. They not only play a role in early immune surveillance, but also in maintaining durable antitumor responses and therapeutic immunity (143, 144). Characterized by strong proliferation, cytotoxicity, and recall sensitivity, they are essential for long-lasting immune defense. TLS provide a specialized niche that maintains activation, differentiation, and prolonged function of memory T cells. Within TLS, DCs guide naïve T cells toward central memory T (Tcm) cells and effector memory T (Tem) cells through antigen presentation and secretion of cytokines like IL-12 and IL-15 (30). Tcm cells, known for their homing and long-term viability, are enriched in early TLS of tumors like HCC, supporting their involvement in long-term antitumor memory (145).

Tissue-resident memory T (Trm) cells, particularly CD103^+^ CD8^+^ Trm cells, a specialized memory T cell subset, are linked to improved prognosis in several solid tumors, including lung cancer, melanoma, bladder cancer, and ovarian cancer (146–151) (**Figure 1**). Trm cells typically reside locally in the tumor, with enhanced killing capacity and the ability to rapidly respond to local antigen exposure. Notably, Trm cells secrete CXCL13, which recruits B cells and promotes TLS formation and stabilization, bridging adaptive immunity and TLS construction (152, 153). Although Trm cells frequently express exhaustion markers such as PD-1, TIM-3, and CD39, accumulating studies have shown that these markers do not imply complete loss of function. In NSCLC, subsets of Trm cells with high CXCL13 and CD39 expression remain potent effector function, and their infiltration density strongly predicts prolonged recurrence-free survival (128, 152, 154–156). This suggests that Trm cells have functional heterogeneity, regulated by antigen persistence, metabolic status, and inhibitory signals in the TME.

Memory T cells in TLS display distinct roles in autoimmune diseases. In chronic pemphigus, a typical autoimmune disease, scRNA-seq and spatial proteomics have identified CXCL13^+^CD4^+^ memory T cells enriched in TLS, promoting B cell aggregation, maintenance of TLS and autoantibody production (157). These cells show high chemotaxis (CCR7, CXCR5) but low TCR responsiveness and are regulated by Tregs, indicating a homeostatic role in maintenance of TLS (**Figure 1**). However, the function of memory T cells in TLS is not always conducive to antitumor responses. In HPV-positive laryngopharyngeal carcinoma, CD161^+^CD8^+^ Trm cells accumulate in TLS but lack effective interaction with B cells, resulting in immature, suppressed TLS (158). This phenomenon suggests that the effector function of Trm cells may depend on spatial and molecular coordination with B cells, DCs, and Tfh cells. Memory T cells have also shown important responsiveness in tumor immunotherapy. For example, a phase II clinical trial of malignant pleural mesothelioma revealed that combined PD-L1 and CTLA-4 blockade effectively activated CD57^+^ memory T cells, which enriched in TLS and correlated with improved overall survival and immune response (159). CD57^+^ T cells are often regarded as ‘terminally differentiated’ memory cells, and their enrichment suggests that immunotherapy may exert durable effects by targeting memory cells.

Taken together, the colonization, differentiation and activation of memory T cells in TLS are critical for the persistence of antitumor immunity. Distinct memory subsets (Tcm, Tem, Trm) show heterogeneous functions and spatial preferences in TLS. Their interactions with DCs, B cells, Tfh cells, and Tregs determine the immune activity of TLS. Future studies should explore how to modulate the composition, activation state and functional plasticity of memory T cells in TLS to enhance the durability and quality of immunotherapy. Especially, it has important clinical prospects in the reactivation of Trm function, regulation of CXCL13 pathway and reversal of immune exhaustion.

### 2.7 Cytotoxic T cells

CD8^+^ T cells are the main effector cells in tumor immunity, possessing powerful cytotoxic activity to recognize and eliminate MHC-I-expressing tumor cells. Myeloid dendritic cells (mDCs) in TLS activate CD8^+^ T cells through antigen presentation and co-stimulatory signals like CD80/CD86, prompting differentiation into Th1-like and cytotoxic phenotypes (88, 160). Remarkably, TCF1^+^ CD8^+^ T cells are enriched in nascent TLS, where they not only support TLS development but also adopt a stem-like state (161). Mechanistically, the TLS niche sustains this stem-like pool through CXCL13-CXCR5-mediated retention and co-stimulatory signals from B cells and MHCII^+^ APCs (162). In melanoma and genitourinary cancers, TCF1^+^ CD8^+^ T cells are frequently co-located in TLS with B cells or MHCII^+^ APCs, which associated with better treatment response and survival (17, 163–165) (**Figure 1**). Transcriptionally, the TCF1-BATF-IRF4 axis maintains the progenitor state and prevents terminal exhaustion, while the local microenvironment provides IL-15 and Tfh-derived IL-21 (as described in Section 2.1) to support survival and counteract exhaustion (18, 57, 63, 117). Mature TLS harbor a higher frequency of ICI-sensitive TCF1^+^PD-1^+^CD8^+^ T cells relative to terminally exhausted TIM-3^+^PD-1^+^ counterparts, thereby favoring favorable responses to immune checkpoint blockade (163, 164, 166). The diversity of this T cell stem-like lineage is not restricted to the CD8^+^ population. EOMES^+^ CD4^+^ T cells differentiated from TCF1^+^ CD4^+^ stem-like T cells have also been found in TLS in autoimmune vasculitis. These cells have cytotoxic potential and can directly mediate immune injury in target tissues, suggesting that the cytotoxic potential of T cells can extend beyond classical phenotypic classifications (167).

Further studies have shown that CD8^+^ T cells in the TME not only perform cytotoxic functions but may also promote TLS formation and maturation by secreting CXCL13 to recruit B cells. TGF-β can upregulate CXCL13 expression in CD8^+^ T cells, leading them to exhibit functional characteristics similar to Tfh cells and contribute to humoral immunity (36, 37, 147, 152, 168). In colorectal and ovarian cancers, some CD8^+^ T cells differentiate into follicular-like cytotoxic T (Tfc) cells expressing CXCR5 and CD40L, enabling them to assist B cell activation and enhance antibody responses^(1)^. Exogenous CXCL13 signals can markedly drive the expansion and maturation of Tfc cells, whose abundance in tumors correlates strongly with improved outcomes (169, 170). However, the function of CD8^+^ T cells in TLS is also limited by various immunosuppressive signals. Treg cells and their secreted factor TGF-β, can induce the dysfunction of CTLs and weaken their effector capacity. However, this immunosuppressive effect can be partially counteracted in the presence of a dominant number of CTL, maintaining a certain degree of antitumor activity (23). Targeting these inhibitory pathways represents a promising avenue in modern cancer immunotherapy. For example, engineered CCR1^+^/TGFBR2^+^ nanovesicles were designed in a SMAD4-deficient colorectal cancer mouse model with high TGF-β levels, simultaneously targeting the CCL9/CCR1 chemotactic axis and neutralizing TGF-β, effectively rejuvenating CTLs within TLS and boosting antitumor immune responses (171).In addition to molecular regulation, the organization of TLS also acts an important role in CD8^+^ T cells behavior. Orderly arranged collagen fibers can enhance CD8^+^ T cell migration and infiltration, boosting targeting and killing potential (172). Therefore, regulation of TLS matrix structure, particularly collagen fiber alignment, may become a new strategy to optimize immune cell function.

In cancer therapy, the activation status of CD8^+^ T cells is closely linked to treatment response. For instance, Gemcitabine-cisplatin (GP) therapy induces the upregulation of MHC-I expression in tumor cells by activating STING - mediated type I interferon signaling pathway, which promotes the massive expansion of CD8^+^ CTLs in TLS, improving the antitumor response and prognosis of nasopharyngeal carcinoma (173). In addition, Nanovaccine-based approaches further accelerate CTLs initial activation and deployment within TLS by promoting antigen presentation and type I interferon release in lymph nodes or tumors (174). Future immunotherapeutic strategies should more thoroughly account for CD8^+^ T cells activation pathways, spatial organization, inhibitory signal intervention, and functional programming within TLS to enhance the depth and durability of antitumor responses.

## 3 Synergistic roles of TLS and T cells in tumor immunity

TA-TLS and tumor-infiltrating lymphocytes (TILs) closely collaborate in regulating antitumor immunity, which has become a research hotspot in recent years. TLS is not only an ectopic immune activation center in the TME, but also an important site for T cell differentiation, activation, migration and sustained response. Numerous studies indicate that distinct T cell subtypes in TLS may either promote or suppress tumor immunity. The abundance, composition, and activation status of T cells are closely correlated with tumor type, immunotherapy response, and clinical prognosis. Thus, the functional state of TLS and T cell populations is increasingly viewed as a potential predictor of clinical outcomes in a variety of cancers.

### 3.1 Biomarkers of clinical prognosis

The role of TLS varies across different disease states, and their impact on prognosis is disease-specific. The presence of TLS in breast cancer correlates strongly with lower tumor invasiveness and metastatic capacity (175, 176). The differences in the composition and characteristics of T cells and B cells in TLS also affect patients’ treatment response and survival prognosis (177). Tfh cells, as central regulators within TLS, show positive correlations with clinical benefit in multiple cancers, including breast cancer (11, 14, 24, 37, 58), CRC (178, 179), NSCLC (147, 180, 181), HNSCC (182, 183), melanoma (184), and ccRCC (5). This benign prognostic correlation is particularly prominent when Tfh cells and B cells form spatial co-localization in TA-TLS (6, 51–54, 185). CXCL13 and IL-21, secreted by Tfh cells, not only mediate B cell activation and GC development but also associate with favorable clinical outcomes, making them potential biomarkers in CRC (178). CXCL13 can be detected by ELISA, while Tfh cells require mIHC—the former is convenient, whereas the latter provides richer spatial information but demands higher technical expertise. Trm cells are primarily applicable for relapse risk assessment in lung cancer, melanoma, and bladder cancer, with infiltration density significantly correlated with relapse-free survival (146, 148). Unlike Tfh cells and CXCL13 which mainly predict therapeutic efficacy, Trm cells more directly reflect local tissue-resident immune memory and are better suited for long-term disease control assessment.

Immunosuppressive markers present more complex prognostic implications. Treg infiltration correlates with poor prognosis across hepatocellular carcinoma, lung cancer, and breast cancer, serving as a marker of immune-cold tumors (23, 108, 110), although its detection is straightforward with conventional IHC, this approach lacks TLS specificity. The Tfr/Tfh ratio more precisely reflects immune balance within TLS; a high ratio is associated with immunosuppressive TLS and poor prognosis in breast cancer, lung adenocarcinoma, and cholangiocarcinoma (23, 24, 141, 142), yet its detection requires mIHC or scRNA-seq for simultaneous Tfh/Tfr quantification, imposing substantial technical demands. An integrated approach combining multiple markers may offer more comprehensive prognostic guidance in future clinical practice.

### 3.2 Predictive value for treatment response

TLS are strongly related to immunotherapeutic responsiveness in various solid tumors, including melanoma (6, 51), ccRCC (5, 51), and HNSCC (7). The presence of TLS often indicates a better therapeutic response to ICIs. Research on neoadjuvant therapy for breast cancer further indicates that TLS and their biological characteristics (such as genetic characteristics of Tfh cells, expression level of CXCL13, and high-density T or B cells infiltration) predict higher pathological complete response (pCR) rates and improved prognosis (37, 186–188). Besides, some studies have linked TLS to an increased risk of immune-related adverse events (irAEs), suggesting a potential dual role of TLS in immunotherapy (34, 189).

While ICIs yields modest results in advanced STS, patients with pre-treatment TLS enrichment show notably better responses to PD-1 blockade and longer survival outcomes (124). This discovery supports the TLS as predictive biomarkers to guide patient stratification, especially in ICIs-based therapeutic settings. At the T cells level, specific subsets of Trm cells, such as CXCL13^+^CD103^+^CD8^+^ TRMs, are highly active in TLS-rich areas and are considered potential predictors of the effect of ICIs (190). Moreover, recent studies have pointed out that T cell exhaustion is not a single “functional exhaustion” state, but rather a precursor state of immune activation. For instance, a positive feedback network may form between PD-1^+^CXCL13^+^ exhausted T cells and TLS at the spatial and functional levels, jointly participating in the remodeling of the intratomoral immune microenvironment and immune activation (191). In colorectal tumors, CD8^+^CXCR5^+^ Tfc cells have shown strong associations with checkpoint molecule levels and increased ICIs responsiveness, positioning them as promising immunologic predictors (169). Hence, personalized immunotherapy strategies should account for the spatial localization, cell composition, maturity and T cell state of TLS to better stratify patients and formulate immunotherapy.

## 4 Conclusion and outlook

As a microenvironmental structural unit characterized by organized lymphocyte aggregation in tumors, TLS has been widely observed in various types of malignancies. With the deepening understanding of TME, TLS is increasingly recognized as a novel and specific biomarker, which has shown significant value not only in the prognosis evaluation, but also in the prediction of immunotherapy response. Therefore, TLS is expected to become a key component of next-generation immunotherapy strategies, providing new insights into improving treatment precision and response rates. Future investigations into TLS are primarily focused on two scientific challenges.

### 4.1 Comprehensive dissection of composition and function within TLS

Despite extensive evidence have shown that TLS is generally associated with favorable immune responses and therapeutic outcomes, their biological role varies considerably across tumor types. Intratumoral and peritumoral TLS may have substantial differences in structural composition and immune function (192). Some studies even suggest that the presence of TLS may correlate with tumor progression in specific contexts (193, 194). This kind of “paradoxical” phenomenon may mainly result from high heterogeneity of immune cell populations within TLS. In recent years, accumulating evidence has highlighted that the phenotype, fate, and spatial distribution of T cells in TLS are decisive for their immune function. Beyond classical activated CD8^+^ cytotoxic T lymphocytes and Tfh cells, TLS may also harbor a variety of novel or “non-canonical” T cell subsets. These include stress response T cells (Tstr) (195), tolerant CD8^+^ T cells (196), and “bystander” T cells (75), each characterized by distinct activation profiles, antigen specificity, and functional potential. These subsets may variably contribute to antigen recognition, immune suppression, or modulation of tumor immunity. Moreover, the spatial coupling relationship and functional coordination between DCs and naive CD4^+^/CD8^+^ T cells within TLS remains poorly understood. As professional APCs, the positioning, maturation status and interaction of DCs with T cells in TLS directly affect antigen-specific T cell priming (20). To dissect these complex cellular networks and functional states, future studies should rely on high-dimensional spatial biology technologies, such as multiplex immunofluorescence and CODEX spatial transcriptomics to reveal the dynamic process of cell-cell interactions in TLS from two dimensions of time and space.

### 4.2 Therapeutic strategies targeting TLS induction

Inducing TLS formation to enhance local immune activation has become an important area of research in cancer immunotherapy. Especially for “cold tumors” characterized by strong immune suppression, TLS induction may be a key to converting them into “hot tumors.” Various strategies have been explored to therapeutically induce TLS, including vaccines, ICIs, local cytokine delivery, and biomaterial-based tissue engineering. In terms of tissue engineering strategies, researchers have used biomaterial scaffolds and hydrogels to construct TLS *in vitro*, which are implanted *in vivo* after loading with tumor-associated antigens (TAA) to trigger immune responses. Preliminary studies have shown that TLS-like structures can induce activation of DCs and recruitment of T and B cells. However, clinical validation remains limited, and its widespread application still faces the problems of technical barriers and insufficient immune safety assessment. Meanwhile, the research progress also exposes several key bottlenecks in the current field. First, the lack of mature, stable, and reproducible TLS mouse models hampers standardized validation of TLS-inducing strategies and limits their clinical translation. Current induction methods often suffer from the problems of insufficient TLS maturity or high structural heterogeneity. Therefore, it is urgent to further optimize animal modeling and biomaterials platforms to more realistically simulate the TLS construction process in human TME (109). Another key challenge lies in managing adverse effects from immune interventions. Given the close association of TLS with T cell activation, excessive TLS formation or activation may disrupt immune homeostasis and lead to autoimmune disorders or irAEs. Studies have shown that PD-1 inhibition enhances Tfh cells activity and may increase the risk of autoimmune responses (197, 198). To reduce immunotoxicity, many clinical trials exclude cancer patients with pre-existing autoimmune diseases (199).

Thus, how to effectively avoid irAEs while strengthening anti-tumor immune response is a key issue that needs to be solved in the design of future treatment strategy. This also requires us to further clarify the relationship between ICIs response and toxicity in order to construct safer and more selective mechanisms for TLS regulation.

Taken together, TLS are not only key components of antitumor immunity within the TME, but also represent a novel and promising therapeutic target in the field of immunotherapy. In-depth studies on the dynamic evolution of TLS structure, immune cell heterogeneity, and mechanisms of therapeutic induction will contribute to a comprehensive understanding of the strength and durability of immune responses in different tumor types. In the future, through the precise regulation and intervention of TLS-related mechanisms, it is expected to significantly improve the immune response rate and durability of treatment, which will ultimately translate into higher clinical outcomes and patient survival benefits.
